# Cosmetic Enzymes as Formulation‐Integrated Actives: Mechanisms, Delivery, and Functional Evidence

**DOI:** 10.1111/jocd.71085

**Published:** 2026-07-22

**Authors:** Asmae Bouziani, Ruhan Özel, Ömer Güngör

**Affiliations:** ^1^ Department of Chemistry and Chemical Processing Technology, Advance Vocational School of Hereke Asım Kocabıyık Kocaeli University Kocaeli Türkiye

**Keywords:** cosmetic bioactives, enzymatic exfoliation, enzyme‐based cosmetics, formulation strategies, skin delivery systems

## Abstract

**Background:**

Enzymes are increasingly incorporated into cosmetic formulations as engineered bioactive components whose performance depends on stabilization, delivery, and controlled activity within complex formulation systems, rather than functioning as standalone ingredients.

**Aims:**

This review examines the mechanisms of action of enzymes used in skincare, including proteases, lipases, antioxidant enzymes, and pigmentation‐modulating systems, and evaluates how formulation design influences their functional efficacy.

**Patients/Methods:**

A narrative literature review was conducted, focusing primarily on studies published between 2020 and 2025, with selective inclusion of older foundational references where mechanistic or historical context was required.

**Results:**

Recent advances in recombinant production, encapsulation technologies, and carrier‐based delivery systems have improved enzyme stability and activity control, enabling broader use in exfoliation, antioxidant protection, and tone regulation. Evidence from recent studies indicates that cosmetic outcomes are governed primarily by formulation architecture and delivery efficiency rather than enzyme class alone. However, translation into consistent product performance remains constrained by limited long‐term clinical data, variability in evaluation models, and unresolved challenges in skin penetration and stability.

**Conclusions:**

Current literature supports a shift toward system‐level design in which enzymes operate as integrated components of engineered cosmetic formulations. Bridging formulation innovation with standardized clinical validation will be essential for establishing robust functional evidence and advancing enzyme‐based cosmetic technologies.

## 1 Introduction

Bioactive materials, particularly enzymes, have attracted increasing attention in cosmetic science due to growing demand for natural, high‐performance, and sustainable products. Enzymes are defined as biocatalysts extracted from microbial, fungal, and plant sources [1, 2]. Within the cosmetics industry, enzymatic ingredients are used across a broad range of functions, including exfoliation, antioxidant protection, anti‐aging, and depigmentation [2]. Their use generally aligns with green chemistry principles, as they are biodegradable and substrate‐selective [3].

Among cosmetic enzymes, proteases are widely employed for exfoliation [4], lipases for sebum regulation [1], tyrosinase inhibitors for pigmentation control [5], and antioxidant enzymes, such as superoxide dismutase (SOD), for protection against oxidative skin damage [6]. Despite these established applications, incorporating enzymes into commercial cosmetic products is still challenging. These constraints are due to enzyme instability, limited skin permeability, and potential allergenicity [7]. Thus, the development of advanced stabilization approaches and delivery systems has become a central focus of recent formulation research.

From another perspective, conventional cosmetic actives, including strong acids, retinoids, and synthetic antioxidants, are frequently associated with adverse effects such as skin irritation, barrier disruption, and limited long‐term tolerability [8, 9, 10]. Enzymes, by comparison, act through relatively substrate‐specific catalytic mechanisms. This behavior may enable more targeted biochemical interactions at the skin surface and potentially reduce nonspecific irritation under appropriate formulation conditions. Yet, the effective integration of enzymatic actives into cosmetic formulations is still limited by challenges related to stability, controlled activity, skin penetration, and immunogenicity. Advances in recombinant enzyme production, encapsulation technologies, and skin‐responsive delivery systems since 2020 have significantly expanded the functional potential of enzymatic cosmetics. Nevertheless, these developments remain scattered across the literature. Hence, this review critically evaluates enzyme‐based cosmetic actives by integrating mechanistic insights, formulation challenges, and recent technological innovations to identify current limitations and future directions for evidence‐based and sustainable cosmetic applications.

This review primarily focuses on studies published between 2020 and 2025, while selectively including older foundational references where mechanistic or historical context is required.

## 2 Classes of Cosmetic Enzymes: Sources, Functional Specificity, and Limitations

**FIGURE 1:**
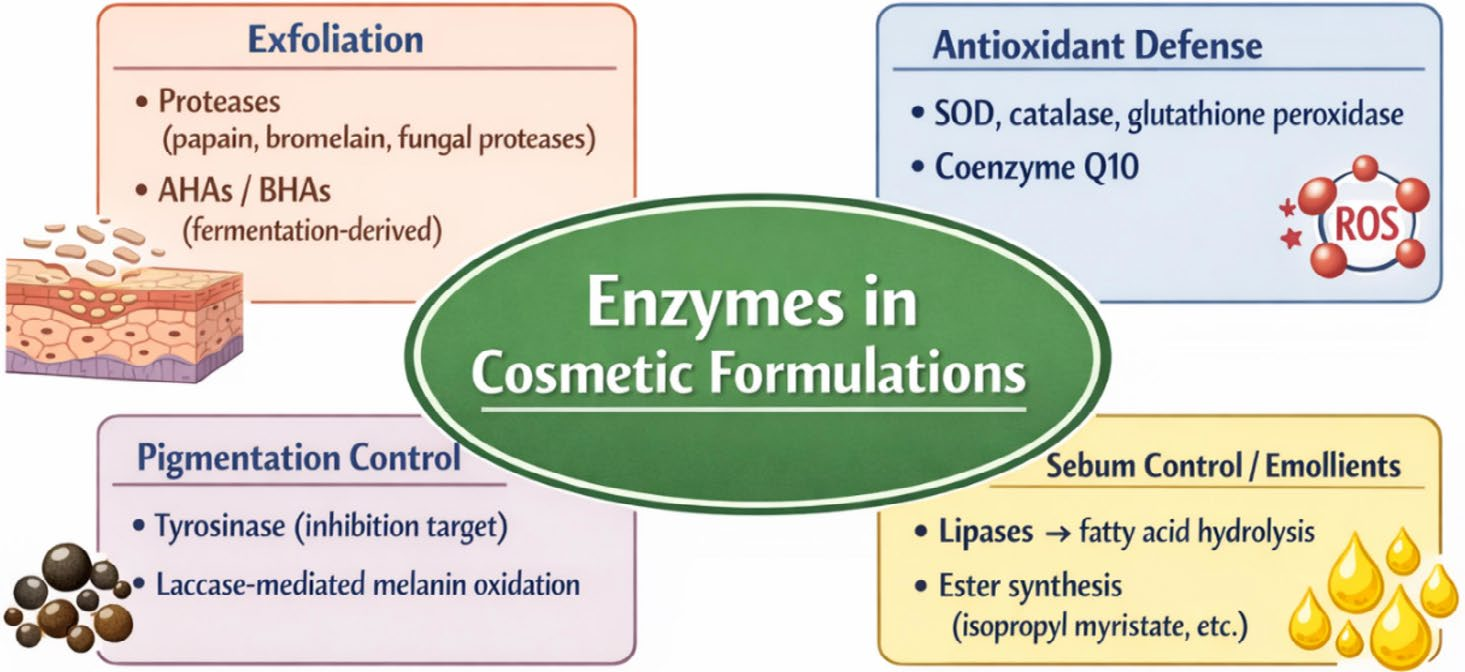
Major enzyme classes used in cosmetic formulations and their primary functional roles. Abbreviations: AHA, alpha‐hydroxy acid; BHA, beta‐hydroxy acid; SOD, superoxide dismutase; ROS, reactive oxygen species.

Modern cosmetics widely use enzymes for their specificity, natural origin, and bioactivity. They are used in exfoliation, antioxidant protection, skin renewal, and pigment regulation (Figure 1). They are extracted from microorganisms, fungi, and plants, or produced through biotechnological processes. It should be noted, however, that not all components discussed within enzyme‐based cosmetic systems are catalytically active enzymes. As classified in Table 1, the field encompasses true enzymes, enzymatic targets, fermentation‐derived or enzyme‐associated actives, and nonenzymatic comparator ingredients; these categories are distinguished consistently throughout the present review.

**TABLE 1:** Classification of enzyme‐related components in cosmetic formulations.

Category	Definition	Representative examples
True enzymes	Catalytically active proteins that directly perform biochemical transformations in cosmetic formulations	Proteases, lipases, superoxide dismutase (SOD), catalase, laccase
Enzymatic targets	Endogenous biological enzymes that are modulated or inhibited by cosmetic actives	Tyrosinase (targeted in pigmentation control)
Fermentation‐derived or enzyme‐associated actives	Compounds produced via enzymatic or fermentation processes but lacking intrinsic enzymatic activity	Alpha‐hydroxy acids (glycolic acid, lactic acid)
Comparator cosmetic actives	Non‐enzymatic ingredients used as benchmarks for evaluating cosmetic efficacy	Coenzyme Q10, kojic acid, arbutin

This classification framework is used throughout the manuscript to distinguish enzyme‐driven mechanisms from non‐enzymatic cosmetic actives and biological targets. Proteases catalyze the hydrolysis of keratin and other structural proteins within the stratum corneum, facilitating desquamation and promoting skin renewal [11, 12]. They are generally incorporated into exfoliating masks and cleansers [10]. Proteolytic enzymes enable selective corneodesmosomal degradation with favorable tolerability profiles when properly formulated. Thus, it reduces the risk of barrier disruption when appropriately formulated and dosed. Nevertheless, their activity remains highly sensitive to formulation parameters, particularly pH and moisture content, leading to inconsistent performance across products. Over the past 5 years, improvements in batch consistency and shelf stability have been achieved primarily through encapsulation strategies and recombinant production technologies [10, 13]. However, standardized clinical comparisons with chemical exfoliants remain limited.

Beyond proteases, lipases are used to hydrolyze lipid‐based impurities, supporting formulations designed for sebum regulation and cleansing [1]. Cellulases contribute to gentle exfoliation and surface smoothing by facilitating the removal of dead skin cells [4]. Hyaluronidases degrade hyaluronic acid and are applied in formulations targeting skin hydration and elasticity modulation [14]. Antioxidant enzymes, including superoxide dismutase (SOD), catalase, and glutathione peroxidase, are primarily incorporated to mitigate oxidative stress and protect against reactive oxygen species‐mediated skin damage [15, 16, 17]. In parallel, alpha‐hydroxy acids (AHAs), often produced via microbial or fungal fermentation, and beta‐hydroxy acids (BHAs), typically synthesized chemically, are widely used as comparator actives in exfoliating formulations rather than enzymatic systems [18, 19].

The definitions adopted for each category are consistent with established descriptions of enzymatic and fermentation‐derived cosmetic ingredients [1, 2, 8, 10]. The following subsections examine, in turn, bacterial and recombinant enzymes, fungal enzymes, and plant‐derived enzymes.

### 2.1 Bacterial and Recombinant Enzymes

Microbial enzymes are derived from bacteria, yeast, and algae. They are widely employed in cosmetic formulations due to their high production yields, scalability, and compatibility with fermentation‐based manufacturing processes [20] and broader industrial reviews of microbial enzyme production and cosmetic compatibility [7, 12]. Advances in biotechnology have accelerated their implementation in cosmetics, enabling more controlled production and improved integration of formulations. Among microbial enzymes, proteases, lipases, carbohydrases, and antioxidant enzymes represent the most frequently utilized functional classes [10, 12, 21]. Representative bacterial sources include Bacillus species, particularly *Bacillus subtilis* and *Bacillus licheniformis* , which are widely exploited for the industrial production of alkaline and neutral proteases used in cleansing and exfoliating systems [12, 21]. Recombinant expression in hosts such as *Escherichia coli* and Bacillus spp. further enables tailored, high‐purity production of subtilisin‐type proteases, bacterial lipases, and engineered antioxidant enzymes, offering improved batch consistency and reduced allergenicity relative to crude extracts [7, 22].

Microbial enzymes, primarily proteases, have been the focus of studies published over the last 5 years [8, 10, 21]. Reported examples include subtilisin‐type and keratinolytic proteases from *Bacillus subtilis* and *Bacillus licheniformis* , together with fungal proteases from Aspergillus and Trichoderma species, which have been evaluated for keratinolytic and exfoliating activity in cosmetic and dermatological contexts [12, 21, 23]. Studies have reported that proteases exhibit consistent functional efficacy in cosmetic applications, both in vitro and ex vivo [24]. This efficacy is enhanced when delivered via encapsulated [24, 25, 26] or recombinant systems [21]. Nonetheless, reported results vary substantially across experimental models [26], enzyme concentrations [9, 20], and exposure periods [23, 26, 27], complicating cross‐study comparisons. Enzymatic exfoliation is increasingly being investigated as a potential alternative or complementary approach to acid‐based exfoliating actives [10, 28]. However, the lack of studies investigating the long‐term effects in controlled clinical trials restricts definitive conclusions about the enhanced or durable effects. Recent literature suggests that the formulation strategy is the principal factor influencing the performance [10, 24, 25, 26]. In particular, proteases are the most investigated due to their applications in exfoliation and consumer‐facing efficacy. While lipases and antioxidant enzymes are more usually assessed in formulation and stability contexts [10, 23, 29].

Microbial enzymes are known for their broad functional diversity. However, this efficacy is more strongly influenced by stabilization and delivery approaches rather than by enzymatic classes. Latest studies have shown that encapsulation, buffering, and recombinant optimization can help overcome intrinsic limitations like pH sensitivity and instability [7, 8, 27, 30]. These findings emphasize the central role of formulation strategy in governing microbial enzyme performance.

### 2.2 Fungal Enzymes

Molds, yeasts, and filamentous fungi produce fungal enzymes. They have been widely investigated for cosmetic applications due to their intrinsic stability in acidic environments and at moderate temperatures [13]. Enzymes derived from genera such as *Aspergillus* and *Trichoderma* are particularly valued for their extracellular secretion profiles and robustness under formulation‐relevant conditions. Experimental studies have demonstrated that fungal hydrolases, including proteases and cellulases, retain catalytic activity at low pH, supporting their suitability for exfoliating and peel‐type cosmetic formulations [31].

Among fungal enzymes, fungal proteases have received the most attention for cosmetic use. Biochemical characterization studies show that these enzymes effectively hydrolyze structural skin proteins involved in corneocyte cohesion. Thus, promoting controlled desquamation and surface smoothing [32]. Their performance in acidic formulations often exceeds that of many bacterial or recombinant microbial proteases, which often require buffering or encapsulation to maintain activity under similar conditions [24].

However, recent literature also highlights limitations associated with fungal enzyme production, particularly regarding reproducibility. Fermentation‐based production is susceptible to strain selection, substrate composition, aeration, and cultivation parameters, leading to variability in enzyme yield and activity between batches [33]. Process‐level investigations have demonstrated that even minor changes in fermentation conditions can significantly alter enzymatic profiles, posing challenges for consistent formulation performance [34].

In addition to enzymes, fungal fermentation is widely used to produce alpha‐hydroxy acids, including glycolic and lactic acids. They are considered the cornerstone actives in chemical exfoliation [35]. These fermentation‐derived acids benefit from established production processes and well‐characterized performance profiles, reinforcing the role of fungi in cosmetic exfoliation strategies.

To address these issues, research has focused on process optimization and downstream control strategies [13, 33, 36]. Statistical optimization of fermentation parameters and improved purification protocols have been shown to enhance batch consistency and functional reliability of fungal enzymes [13, 31, 32, 33]. However, complete standardization remains more challenging than in recombinant microbial systems [31, 32, 33]. In parallel, formulation‐driven approaches, such as buffering systems and encapsulation, are increasingly explored to stabilize fungal enzymes during storage and application [37, 38]. Although less extensively than for microbial recombinant enzymes.

In summary, recently published research suggests that fungal enzymes offer natural robustness and compatibility with acidic cosmetic formulations [39, 40, 41, 42]. However, their broader implementation is limited by production variability and limited tunability [33, 43]. Compared to recombinant microbial enzymes, which allow exact control over enzyme structure and performance via genetic and process engineering [22, 44]. Fungal‐derived enzymes offer stability‐driven advantages but reduced flexibility [45]. Therefore, the current literature supports a balancing role for fungal and microbial enzymes. In which enzyme selection is ever more influenced by formulation context and performance requirements rather than biological origin alone [7, 24, 30].

### 2.3 Plant‐Derived Enzymes

**FIGURE 2:**
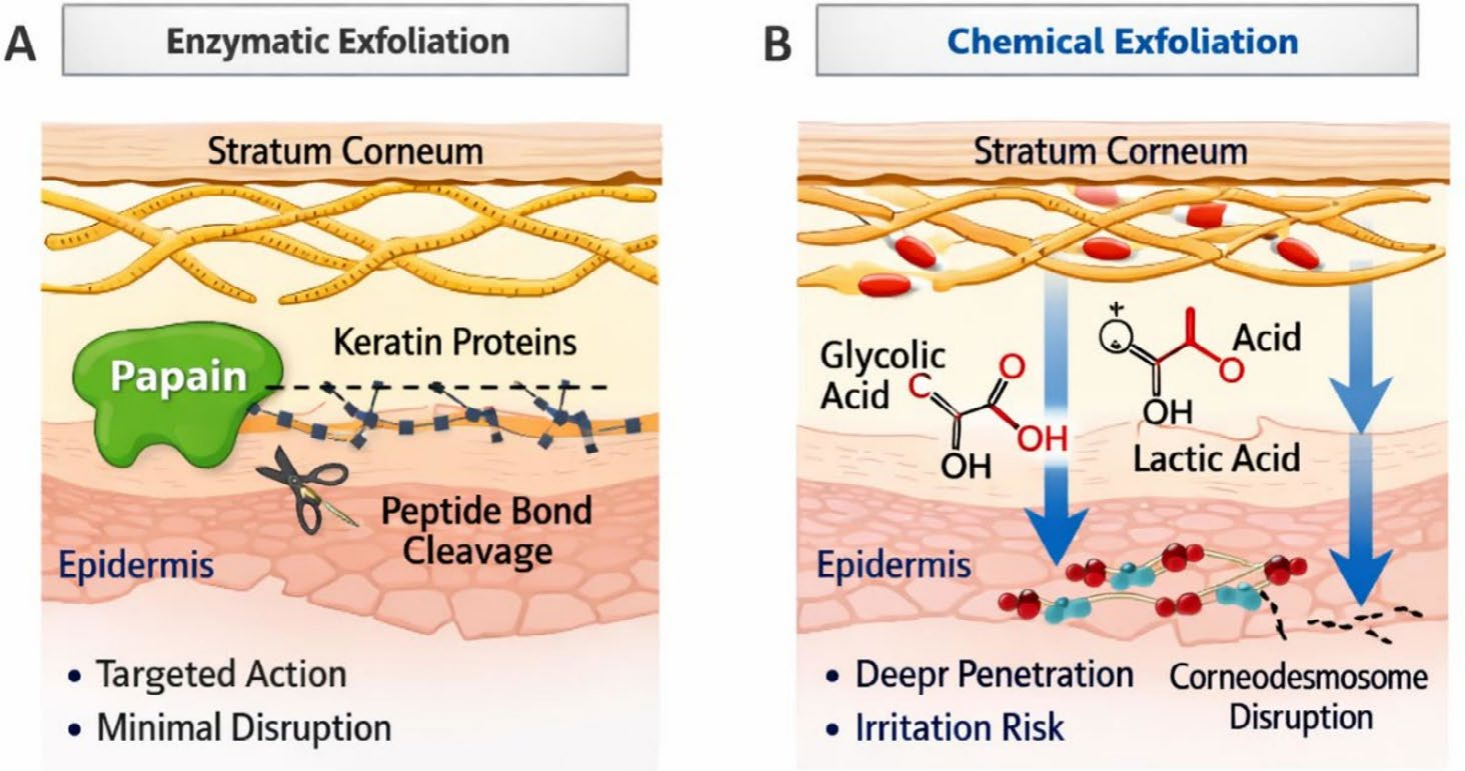
Conceptual comparison between enzymatic exfoliation by plant‐derived proteases and chemical exfoliation by alpha‐hydroxy acids, highlighting differences in substrate specificity and depth of skin interaction.

Plant‐derived enzymes remain widely used in cosmetic formulations, particularly in products marketed for gentle or sensitive‐skin applications. Among these, papain and chymopapain from *Carica papaya* and bromelain from *Ananas comosus* are the most extensively applied cysteine proteases due to their ability to selectively hydrolyze proteinaceous debris at the stratum corneum surface [10, 46]. Their long history of consumer use and favorable tolerability profiles have contributed to sustained commercial implementation, with their proteolytic and skin‐resurfacing activities and incorporation into commercial cosmetic and cosmeceutical products documented in recent comparative analyses [10, 23, 29]. The fundamental differences between substrate‐specific enzymatic exfoliation and nonspecific chemical exfoliation are schematically illustrated in Figure 2.

Recent studies, however, indicate that the cosmetic performance of plant‐derived enzymes is strongly formulation‐dependent, with enzymatic activity susceptible to pH, temperature, moisture, and the presence of formulation excipients [10, 24, 30]. Analytical evaluations of enzymatic exfoliants have demonstrated substantial variability in measurable proteolytic activity across commercial products. This suggests that enzyme presence alone does not guarantee functional efficacy at the point of use [10]. This highlights a key limitation of plant‐derived enzymes: although their biochemical mechanisms are well understood, their activity retention in finished cosmetic formulations remains inconsistent.

To address these limitations, research published in the last 5 years increasingly emphasizes delivery‐ and stabilization‐driven strategies. Encapsulation approaches, including liposomes, niosomes, and chitosan‐based nanoparticles, have been shown to improve the stability, controlled release, and functional persistence of papain and bromelain in topical systems [24, 25, 30]. These studies demonstrate that formulation architecture exerts a greater influence on performance than enzyme origin, reinforcing a formulation‐first paradigm consistent with trends observed for microbial enzymes.

Beyond exfoliation, plant‐derived proteases have also been investigated for anti‐inflammatory and wound‐healing‐related bioactivities. These behaviors support their multifunctional potential in cosmetic and dermatological contexts [10, 29]. However, most recent evidence remains limited to in vitro, ex vivo, or short‐term application models. Furthermore, there is a notable scarcity of controlled, long‐term clinical trials published in the last 5 years. As a result, claims regarding sustained cosmetic benefits or enhancement over other enzymatic systems remain insufficiently substantiated.

On the other hand, recombinant microbial enzymes provide superior tunability at the molecular level, allowing precise adjustments to their functional properties. In contrast, plant‐derived enzymes remain largely limited by the constraints of their native structures. As a result, performance optimization in plant‐derived enzymes depends primarily on formulation approaches rather than genetic or bioprocess engineering [7, 24, 30].

Taken together, the recent literature supports the view that plant‐derived enzymes play a complementary role in enzymatic cosmetic formulations. Their strengths lie in mild exfoliation, consumer familiarity, and favorable tolerability. Their limitations are primarily associated with stability, activity control, and limited contemporary clinical validation [10, 24, 30]. As with fungal and microbial enzymes, current evidence indicates that formulation context and performance requirements increasingly outweigh biological origin alone in determining enzyme selection for cosmetic applications [7, 24]. A comparative overview of enzyme categories, sources, formulation strategies, and levels of evidence is summarized in Table 2. Although mechanistic and formulation‐focused studies dominate the current literature, a limited number of human and clinically oriented investigations have evaluated the cosmetic performance of enzyme‐based formulations.

**TABLE 2:** Summary of enzymes used in cosmetic applications.

Enzyme category	Source	Representative enzymes	Primary cosmetic function	Key formulation strategy	Evidence level	References
Microbial enzymes	Bacteria, yeast, algae	Proteases, lipases, cellulases, hyaluronidases	Exfoliation, sebum control, hydration modulation	Encapsulation (liposomes, polymeric carriers), recombinant production	In vitro/limited clinical	[10, 13]
Fungal enzymes	*Aspergillus*, *Trichoderma*	Fungal proteases, fermented AHAs	Enzymatic exfoliation, skin smoothing	Fermentation optimization, pH‐controlled formulations	In vitro/ex vivo	[9, 13]
Plant‐derived enzymes	*Carica papaya*	Papain, chymopapain	Gentle exfoliation, anti‐inflammatory effects	Stabilized enzyme blends, low‐dose formulations	Clinical/consumer use	[10]
Antioxidant enzymes	Microbial or recombinant	SOD, catalase, glutathione peroxidase	ROS neutralization, anti‐aging	Liposomal delivery, antioxidant co‐formulation	In vitro/ex vivo	[15, 17]
Coenzymes	Biosynthesized	Coenzyme Q10	Antioxidant protection, collagen support	Nanoencapsulation, oil‐phase solubilization	Clinical	[9, 47]
Pigmentation‐related enzymes	Fungal/mammalian (targeted)	Tyrosinase (inhibition target)	Skin brightening, tone correction	Controlled‐release inhibitors, antioxidant combinations	In vitro/limited clinical	[9, 48]
Oxidoreductases	Fungal	Laccase	Detoxification, safer dye formulations	Enzyme replacement of H_2_O_2_, mild oxidizing systems	Industrial/in vitro	[49, 50]
Lipolytic enzymes	Microbial (biotech fermentation)	Lipases	Cleansing, emollient synthesis, sebum regulation	Immobilized enzymes, surfactant‐free systems	Industrial/in vitro	[1, 51]
Comparator or fermentation‐derived actives	Fermentation‐derived or synthetic	Glycolic acid, lactic acid	Chemical exfoliation, acne treatment	Buffered delivery systems	Clinical	[18, 19]

## 3 Functional Roles of Enzymes in Skin Physiology

**FIGURE 3:**
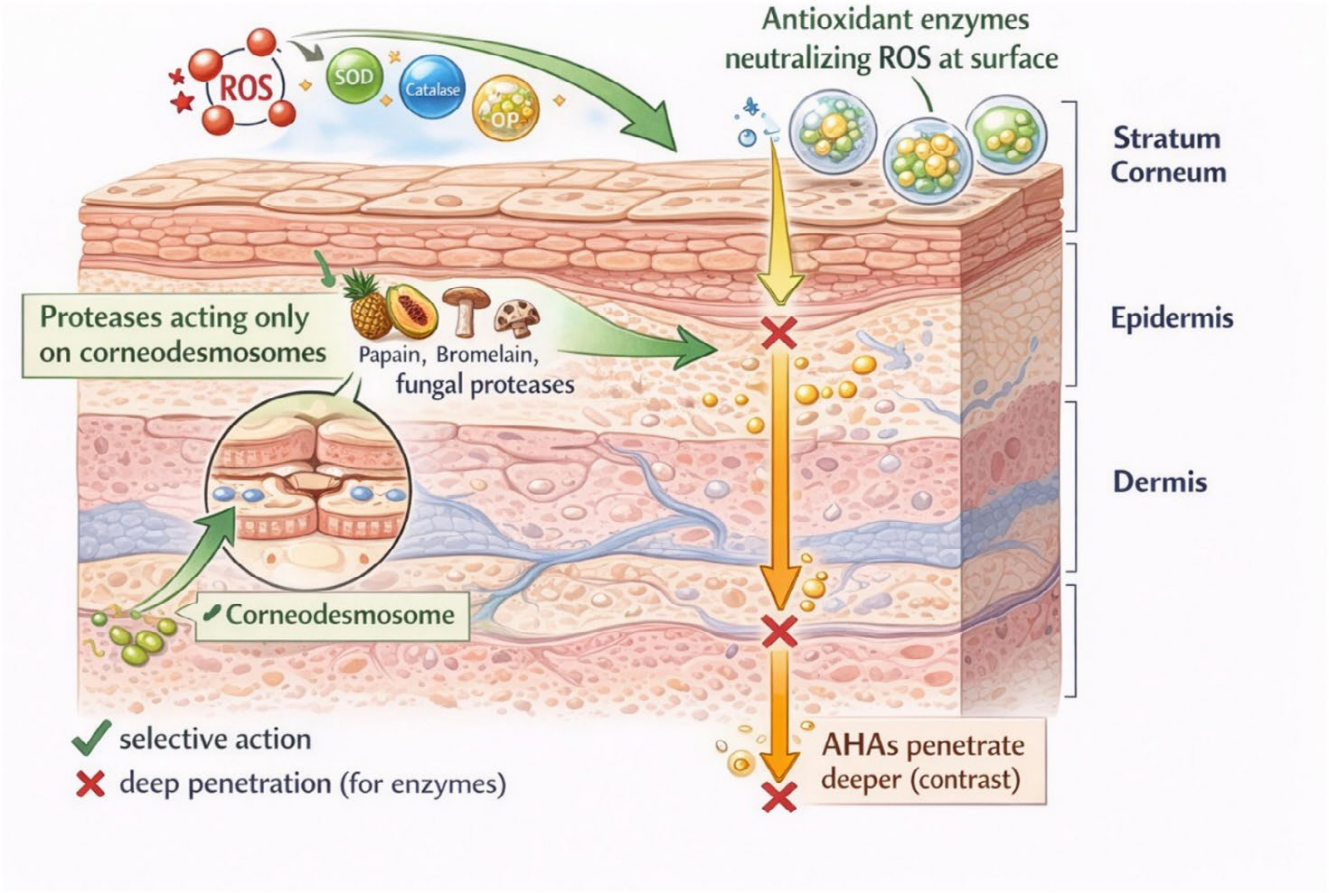
Functional roles of cosmetic enzymes in skin exfoliation, antioxidant defense, pigmentation regulation, and lipid modulation.

Enzymes exert their cosmetic benefits through specific biochemical actions that target skin layers and cellular processes [8]. Their effectiveness depends on factors such as substrate specificity, pH, delivery system, and concentration. Because most cosmetic enzymes are large, hydrophilic macromolecules, typically exceeding the size threshold for efficient passive diffusion across the intact stratum corneum, they are generally unable to penetrate deeper viable epidermal or dermal layers in their native form [15, 24]. Consequently, their cosmetic activity is largely confined to the skin surface and the outermost corneocyte layers unless carrier‐based delivery systems are employed, which frames the functional roles summarized below as predominantly surface‐ and barrier‐associated processes. An overview of the primary enzymatic functions in skin physiology is summarized in Figure 3.

### 3.1 Exfoliating Enzymes

Proteases (e.g., papain and fungal proteases) function as enzymatic exfoliants, whereas alpha‐hydroxy acids (AHAs) and beta‐hydroxy acids (BHAs) are non‐enzymatic chemical exfoliants that also promote desquamation of the stratum corneum. This results in smoother, brighter skin and enhanced cell turnover [4, 52].

Papain and chymopapain hydrolyze peptide bonds in keratin (Equation 1) and other epidermal proteins, facilitating the removal of dead skin cells and promoting a smoother skin texture. Their proteolytic activity has also been investigated for potential wound‐healing and anti‐inflammatory effects, making them ideal for facial masks, peels, and enzymatic exfoliants [46]. The incorporation of papain and related proteolytic enzymes into facial masks, peel‐type formulations, and other exfoliating cosmetic systems has been further evaluated in recent cosmetic and dermatological studies [10, 23].

(1) $$\text{Keratin}+H_{2}O\;\overset{\text{Papain}/\text{Chymopapain}}{\to }\;\text{Peptide}\to \text{Amino Acids}$$

The activity of these enzymes is influenced by pH, temperature, and the presence of inhibitors. Optimal efficacy is achieved at slightly acidic to neutral pH and moderate temperatures [46]. Although generally well tolerated, patch testing is recommended for individuals with sensitive or reactive skin. Compared with chemical exfoliation, enzymatic exfoliation may provide greater substrate selectivity and improved tolerability under appropriate formulation conditions. However, robust comparative clinical evidence remains limited, and direct long‐term comparisons with conventional chemical exfoliants remain insufficiently established [49].

### 3.2 Antioxidant Enzymes and Related Antioxidant Cosmetic Actives

**FIGURE 4:**
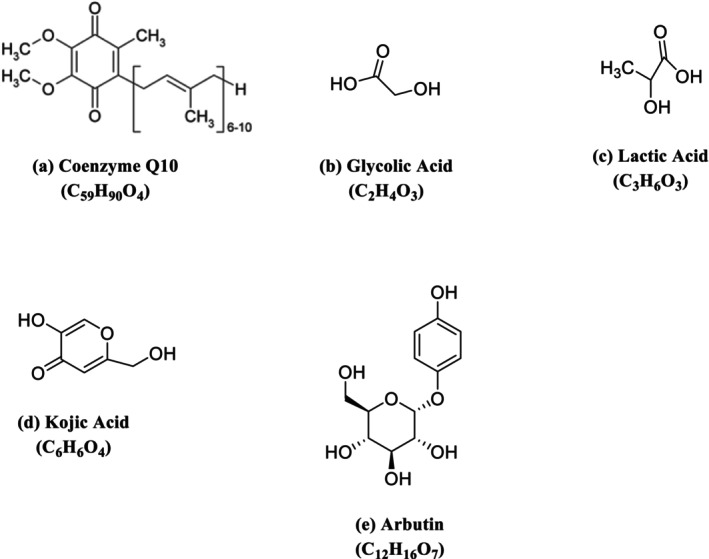
Chemical structures of representative low‐molecular‐weight cosmetic actives used as benchmarks in enzyme‐based formulations: (a) coenzyme Q10, (b) glycolic acid, (c) lactic acid, (d) kojic acid, and (e) arbutin.

Enzymatic antioxidants such as superoxide dismutase (SOD), catalase, and glutathione peroxidase, together with related non‐enzymatic antioxidant actives such as coenzyme Q10 (CoQ10), are used to reduce oxidative stress [15, 16, 53]. This protects skin lipids, proteins, and DNA from UV‐induced damage, supporting anti‐aging and barrier function [15, 17, 47]. Moreover, low‐molecular‐weight compounds are commonly used as a reference or benchmark ingredient in cosmetic formulations. Examples include alpha‐hydroxy acids used for chemical exfoliation and small‐molecule depigmenting agents targeting melanogenesis. Representative chemical structures of widely used benchmark compounds are shown in Figure 4.

*Coenzyme Q10*: Coenzyme Q10 is frequently discussed alongside enzymatic antioxidants because of its central role in cellular redox balance, although it is not itself an enzyme. 2,3‐dimethoxy‐5‐methyl‐6‐decaprenyl‐benzoquinone, or ubiquinone (Figure 4a), is a lipid‐soluble benzoquinone found in the mitochondrial membrane, where it plays a critical role in ATP production and redox cycling. Its antioxidant activity in the skin improves elasticity and firmness, lowers lipid peroxidation, and guards against UV‐induced damage [53]. It has been demonstrated that CoQ10 improves skin resilience and smoothness [47]. However, oxidative instability and poor water solubility restrict its use. Modern formulations use liposomal carriers, nanoencapsulation, and co‐delivery with stabilizing agents such as hyaluronic acid and vitamin E to address these problems [15, 47].

*SOD* is a natural enzyme that serves as our body's primary defense against oxidative stress. It protects our cells from damage caused by free radicals. SOD is an enzymatic antioxidant. It constitutes the main line of defense against free radicals in the cell. It catalyzes the conversion of superoxide radical (O_2_•‐) into molecular oxygen (O_2_) and hydrogen peroxide (H_2_O_2_) (Equation 2) [54, 55, 56, 57]. In this reaction, two superoxide molecules accept two protons to form hydrogen peroxide and molecular oxygen [58]. Thus, it reduces the potentially harmful superoxide anion.

(2) $$2O_{2}^{\cdot -}+2H^{+}\;\overset{\mathrm{SOD}}{\to }\;H_{2}O_{2}+O_{2}$$

In cosmetic formulations, SOD is primarily incorporated into anti‐aging and photoprotective products due to its ability to mitigate oxidative stress [59, 60, 61]. However, its effectiveness remains strongly dependent on formulation strategy, stability, and delivery efficiency.

Catalase is an antioxidant enzyme that decomposes hydrogen peroxide into water and molecular oxygen (Equation 3) [16]. Thus reducing oxidative stress associated with photoaging and inflammation [54]. In cosmetic formulations, catalase is generally used in combination with other antioxidants [16]; however, its effectiveness, like that of other enzymatic antioxidants, is strongly dependent on formulation stability and controlled delivery.

(3) $$2\;H_{2}O_{2}\;\overset{\text{Catalase}}{\to }\;2H_{2}O+O_{2}$$

While enzymatic antioxidants exhibit high catalytic efficiency, their cosmetic performance is primarily limited by delivery and stability rather than intrinsic activity.

Studies published in the last 5 years consistently demonstrate that the cosmetic effectiveness of antioxidant enzymes is primarily affected by delivery efficiency rather than intrinsic enzymatic activity [62, 63]. Encapsulated SOD and catalase systems outperform free enzymes in oxidative stress models. However, clinical evidence remains primarily short‐term and biomarker‐based. This gap highlights a recurring limitation in the field, where formulation innovation advances faster than clinical validation [15].

### 3.3 Skin Brightening and Pigmentation Control

Hyperpigmentation disorders such as melasma, age spots, and post‐inflammatory pigmentation arise from the excessive or uneven distribution of melanin in the skin. Unlike the proteases and antioxidant enzymes discussed above, tyrosinase is not used as a cosmetic enzyme but instead represents a key biological target in melanogenesis; cosmetic formulations therefore focus on inhibiting its activity through competitive or allosteric mechanisms to even skin tone and reduce hyperpigmentation [9].

These pigmentation concerns have made the controlled modulation of melanin synthesis, principally through tyrosinase, a central objective in cosmetic brightening and tone‐correction formulations [64].

Tyrosinase is a copper‐containing oxidase that catalyzes two crucial reactions in melanin biosynthesis. The hydroxylation of tyrosine to L‐DOPA (L‐3,4‐dihydroxyphenylalanine) and oxidation of L‐DOPA to dopaquinone [48]. These reactions initiate a series of chemical transformations that result in the formation of eumelanin and pheomelanin (Equation 4). Overactivity of tyrosinase can lead to localized or diffuse hyperpigmentation [48].

(4) $$\text{Tyrosine}\;\overset{\text{Tyrosinase}\;}{\to }\;L-\text{DOPA}\;\overset{\text{Tyrosinase}\;}{\to }\;\text{Dopaquinone}$$

**FIGURE 5:**
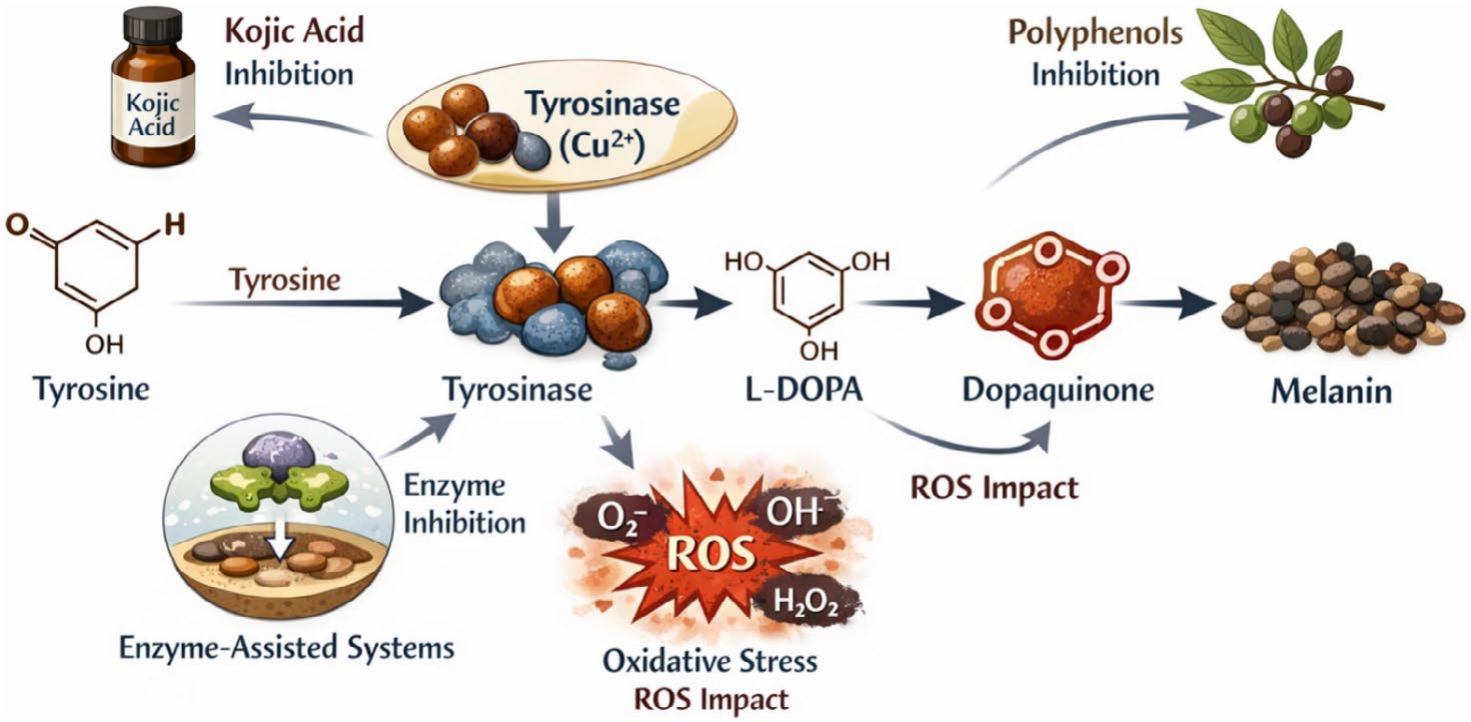
Simplified melanogenesis pathway showing tyrosinase‐catalyzed steps and cosmetic intervention points.

To contextualize enzymatic and non‐enzymatic depigmenting strategies, the key steps of melanogenesis and principal intervention points targeted by cosmetic actives are summarized in Figure 5.

To address pigmentation concerns, tyrosinase inhibitors are often incorporated. Conventional depigmenting agents such as kojic acid and arbutin inhibit tyrosinase activity through different mechanisms, but are often associated with irritation or limited long‐term tolerability [48, 65]. Some cosmetic products also use enzyme delivery systems to improve skin permeability and reduce irritation, particularly when used with traditional depigmenting agents [66, 67].

Recent research has explored recombinant tyrosinase variants, enzyme encapsulation, and co‐formulation with antioxidants to boost efficiency while lessening adverse effects [5, 48]. These approaches support the development of safer and more effective depigmenting products.

*Laccases*: In recent years, there has been a significant increase in the number of laccase‐related patents in the cosmetic industry [50]. Laccases have oxidizing ability. Therefore, they can eliminate harmful chemicals such as H_2_O_2_ and phenylenediamine [49]. Hydrogen peroxide (H_2_O_2_) and phenylenediamine are hazardous compounds found mainly in hair dyes and can cause carcinogenic and allergic reactions. Laccase‐based hair dyes are much less irritating, and since they contain laccase instead of hydrogen peroxide found in regular hair dyes, they are easier to use than hair dyes [68]. Therefore, laccases are used as valuable biocatalysts in some cosmetic preparations (e.g., deodorants, perfumes, toothpastes, hair dyes, and soaps) [49]. Laccase enzymes can also be used in cosmetic products as skin whiteners [69]. Compared with some conventional tyrosinase inhibitors, enzyme‐assisted depigmenting strategies may support more gradual modulation of pigmentation and could offer favorable tolerability profiles; however, comparative long‐term clinical evidence remains limited.

Recent investigations have increasingly explored indirect modulation of melanogenesis as a potentially better‐tolerated alternative to strong tyrosinase inhibition strategies, reflecting concerns about safety and tolerability. Comparative studies using reconstructed epidermis and short‐term in vivo models suggest that enzyme‐assisted systems yield more gradual, yet sustained, pigmentation improvement [4, 8, 21, 70]. Although with modest effect sizes. Nevertheless, heterogeneity in pigmentation models and evaluation methods continues to hinder meaningful cross‐study comparison.

### 3.4 Lipases in Cleansing, Emollient Production, and Delivery Systems

Lipases are widely used in cosmetics for their ability to hydrolyze triglycerides and esters, aiding sebum control, skin cleansing, and emollient production. They catalyze the breakdown of lipid‐based impurities in clogged pores, facilitating oil removal and contributing to sebum management, which may help reduce acne formation [1]. This makes them valuable in formulations targeting oily or acne‐prone skin.

In emulsions, lipases enhance ingredient penetration and enable surfactant‐free cleansing systems. They have also been employed in the enzymatic synthesis of esters, such as isopropyl myristate, isopropyl palmitate, and 2‐ethylhexyl palmitate, which serve as emollients in skincare products such as sunscreens, bath oils, and creams. Companies like Unichem International (Spain) use immobilized Rhizomucor miehei lipase for the biocatalytic production of these esters, citing improved product quality despite slightly higher costs [71, 72]. Lipases also participate in the enzymatic synthesis of mono‐ and diacylglycerols, which function as biodegradable surfactants in personal care and fragrance industries [73]. Their application has expanded to the enzymatic preparation of water‐soluble retinoid derivatives, enhancing vitamin A's compatibility and delivery in skincare [51].

Additionally, lipases are found in niche formulations such as anti‐obesity creams, hair waving agents, and topical fat metabolism enhancers, reflecting their multifunctional value in dermatological and cosmetic innovations [74]. In anti‐obesity or so‐called “slimming” topical formulations, lipases are proposed to assist the local hydrolysis of stored triglycerides, often in combination with methylxanthines such as caffeine, to support the appearance of reduced subcutaneous fat, although robust clinical evidence for a true lipolytic effect through intact skin remains limited. In hair‐care applications, lipase‐ and other enzyme‐assisted systems have been explored as milder alternatives to thioglycolate‐based permanent‐waving agents, aiming to modify hair structure with reduced cuticle damage. Likewise, lipases incorporated into formulations targeting topical fat metabolism are intended to act on cutaneous and sebaceous lipids rather than to induce systemic effects. Across these niche uses, reported benefits are derived predominantly from in vitro or small‐scale studies, and their cosmetic relevance remains constrained by the limited skin penetration and stability of lipolytic enzymes [51, 71, 74].

Despite this functional versatility, the cosmetic performance of lipase‐based systems is governed less by intrinsic catalytic activity than by formulation‐level factors. Lipases are sensitive to pH, water activity, surfactants, and oxidation, so maintaining controlled and reproducible activity within finished products typically requires immobilization, encapsulation, or stabilizing excipients. Their relatively large molecular size and hydrophilic character further limit cutaneous penetration, confining most activity to the skin surface and sebaceous openings. From a manufacturing perspective, immobilized or recombinant lipases can increase production costs and add downstream‐processing complexity, while batch‐to‐batch variability and shelf‐life constraints remain practical hurdles. Consequently, the successful incorporation of lipases into cosmetic formulations depends on balancing enzymatic control, delivery efficiency, stability, and cost rather than on enzyme selection alone [7, 51, 75].

## 4 Formulation Challenges and Technological Solutions

**FIGURE 6:**
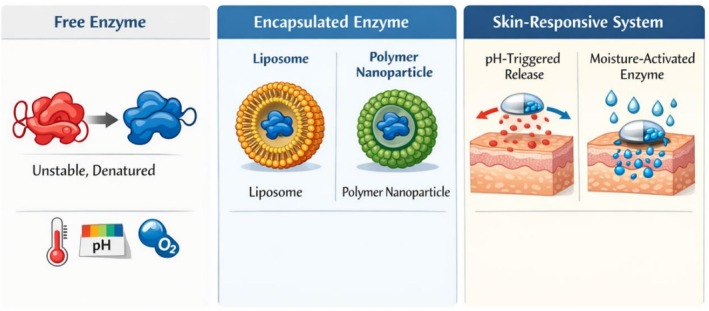
Formulation and delivery strategies used to improve enzyme stability, activity control, and skin performance.

Despite their biological advantages, enzymes pose formulation challenges that must be overcome to ensure efficacy, safety, and shelf stability in cosmetic products [10, 24, 26]. This section defines key technical issues and the strategies used to fix them. Key formulation and delivery strategies employed to enhance enzyme stability and control activity are summarized in Figure 6.

The principal formulation challenges affecting enzyme‐based cosmetic products, including stability, delivery, formulation compatibility, safety, and shelf life, are summarized in Table 3. These constraints form the basis for the formulation‐driven performance trends and comparative outcomes discussed in the following sections.

**TABLE 3:** Formulation challenges and mitigation strategies for enzyme‐based cosmetics.

Challenge category	Key issues	Formulation strategies	References
Enzyme stability	Denaturation by heat, light, extreme pH; oxidative degradation	Encapsulation (liposomes, nanocarriers, polymer matrices); buffering systems; stabilizers (glycerol, sugars, antioxidants)	[7, 8, 10, 24, 25, 26, 30, 75]
Skin delivery & penetration	Large molecular size; hydrophilicity; surface‐limited activity	Penetration enhancers (ethanol, hyaluronic acid); nanocarriers; enzyme‐derived peptides; proenzyme systems	[10, 24, 25, 26, 30]
Compatibility with complex formulations	Inactivation by surfactants, preservatives, metal ions; processing stress	Compatibility screening; chelating agents (e.g., EDTA); microencapsulation	[8, 10, 24, 27, 30]
Allergenicity & irritation	Immunogenicity risk; over‐exfoliation; barrier disruption	Low‐dose formulations; dermatological patch testing; recombinant enzymes with reduced allergenicity	[8, 15]
Shelf life & packaging	Activity loss during storage; sensitivity to oxygen, light, temperature	Airless and opaque packaging; single‐use formats; freeze‐dried or cold‐chain products	[10, 21, 27, 75]

Despite advances in delivery technologies, the cutaneous penetration of many cosmetic enzymes remains inherently limited by their relatively large molecular size, hydrophilic character, and structural sensitivity [17, 23, 24]. Consequently, most topically applied enzymes are believed to exert their primary activity predominantly at the skin surface or within the stratum corneum rather than in deeper viable epidermal or dermal layers [10, 17, 49]. Although encapsulation systems, carrier technologies, and penetration‐enhancing strategies may improve enzyme stability and superficial bioavailability [23, 24, 27], direct evidence demonstrating substantial deep skin penetration remains limited for many cosmetic enzyme systems. Therefore, the biological effects of enzyme‐based formulations should generally be interpreted within the context of surface‐associated or barrier‐level activity unless deeper tissue delivery has been experimentally confirmed [17, 23].

## 5 Safety, Stability, and Regulatory Considerations

Despite their growing use in cosmetic formulations, enzyme‐based systems present several safety, stability, and regulatory challenges that must be carefully considered during product development. Proteolytic enzymes, particularly papain, bromelain, and ficin, may induce irritation, excessive desquamation, or allergic sensitization in susceptible individuals, especially when used in high concentrations or in leave‐on formulations [10, 28, 49]. Cases of contact hypersensitivity and occupational sensitization associated with certain proteolytic enzymes have also been reported in the literature [28, 49].

The risk of over‐exfoliation represents an additional concern, particularly when enzymatic exfoliants are combined with other active ingredients such as alpha‐hydroxy acids, retinoids, or physical exfoliating systems. Excessive disruption of the stratum corneum may impair barrier integrity and increase transepidermal water loss, irritation, and skin sensitivity [47, 49]. Consequently, formulation parameters, including enzyme concentration, exposure time, pH, and delivery system, must be carefully optimized to balance efficacy and tolerability.

Product format also substantially influences safety outcomes. Rinse‐off formulations generally provide shorter enzyme exposure times and may reduce irritation potential compared with leave‐on products, which require greater formulation control and tolerability assessment [23, 24, 27]. For this reason, patch testing and dermatological compatibility studies remain important during cosmetic product development, particularly for formulations intended for sensitive skin applications [49].

In addition to consumer safety considerations, manufacturing and storage stability remain critical challenges for enzyme‐containing cosmetics. Enzymes are susceptible to denaturation caused by temperature fluctuations, oxidation, moisture exposure, incompatible excipients, and pH instability [17, 23, 52, 57]. Encapsulation technologies, lyophilization strategies, and controlled delivery systems have therefore been increasingly investigated to improve shelf‐life stability and preserve enzymatic activity during storage [23, 24, 27].

From a regulatory perspective, cosmetic enzymes are generally regulated according to ingredient safety rather than pharmacological activity; however, safety documentation, purity control, microbial quality, allergenicity assessment, and stability testing remain essential requirements for commercialization [76, 77]. Regulatory expectations may also vary depending on jurisdiction, formulation claims, and intended product use. As enzyme‐based cosmetics continue to evolve, greater standardization of safety assessment methodologies and long‐term tolerability studies will likely become increasingly important.

## 6 Trends and Knowledge Gaps

There has been a noticeable shift in the theoretical and technological approaches to the study of enzyme‐based cosmetic actives, with recent experimental evidence. Studies published between 2020 and 2025 increasingly emphasize formulation‐driven performance rather than just enzyme identification and biological function. The evolution of enzymatic cosmetics from specialized “natural alternatives” to engineered functional systems is reflected in controlled enzymatic activity and consumer safety [75, 78].

The transition from crude enzyme extracts to recombinant and bioengineered enzymes has been one of the most notable trends since 2020. This change provided better purity and reproducibility, as well as a lower risk of allergies [79]. This change has made it easier to comply with regulations and enabled stricter control over enzymatic activity, especially in leave‐on formulations. Encapsulation and carrier‐based delivery methods, such as liposomes, polymeric nanoparticles, and hybrid lipid matrices, have also taken center stage in enzyme formulation strategies and frequently have a bigger impact on efficacy than the origin or class of the enzyme [75, 79, 80, 81].

Another notable trend is the increasing positioning of enzymatic actives as potentially gentler, long‐term skincare approaches rather than rapid‐acting exfoliation systems.

In line with the growing need for cosmetics that are barrier‐preserving and microbiome‐friendly, experimental evidence over the past 5 years has increasingly suggested that enzymes may contribute to gradual exfoliation, sustained antioxidant defense, and modulation of skin physiology [10, 78]. An important change in pigmentation control has been the shift away from aggressive tyrosinase inhibition toward enzyme‐assisted, indirect regulation of melanogenesis, prioritizing safety and tolerability over immediate visible effects [82, 83].

In order to improve stability and expand cosmetic benefits, the literature also shows growing interest in multifunctional, synergistic formulations that combine enzymes with humectants, antioxidants, or biomimetic peptides [80, 84]. This integrative method highlights the growing complexity of enzyme‐enabled cosmetic systems and represents a shift from single‐active formulations.

Even with these developments, there are still a number of important gaps. Most notably, clinical validation continues to lag behind formulation innovation. Relatively few well‐designed, long‐term clinical trials published since 2020 support claims for wrinkle reduction, pigmentation correction, or barrier repair, despite in vitro and ex vivo studies consistently showing promising enzymatic activity [83, 85]. Correlating enzymatic mechanisms with significant cosmetic outcomes is limited by the prevalence of short‐term biomarker studies.

The absence of standardized evaluation procedures is yet another important drawback. It is challenging to make direct comparisons between recent studies because enzyme efficacy is evaluated using widely different models, substrates, concentrations, and exposure durations [78, 86, 87, 88]. Meta‐analysis is hampered by this methodological heterogeneity, which also yields conflicting findings on the superiority of enzymes over traditional cosmetic active ingredients.

Additionally, site‐specific activity and skin penetration are still poorly understood, especially for large, hydrophilic enzymes. Even though advanced delivery systems enhance surface performance, there is still little proof of controlled activation in deeper epidermal layers [75, 81]. This calls into question the biological validity of some claims about enzymes, particularly those related to depigmenting and anti‐aging products.

Lastly, academic studies do not always incorporate safety and regulatory considerations. A gap between lab research and commercial viability is created by numerous recent publications that prioritize mechanistic performance over scalability, batch consistency, or compliance with cosmetic regulatory frameworks [79, 89]. Representative studies, including their formulation strategies, evaluated endpoints, and principal limitations, are summarized in Table 4.

**TABLE 4:** Representative human and clinically oriented studies involving enzymatic and enzyme‐associated cosmetic systems.

Enzyme/active system	Formulation type	Study model/sample size	Duration	Evaluated endpoints	Main outcomes	Major limitations	References
Papain	Enzymatic exfoliating formulations and facial applications	Human cosmetic use reports/limited clinical evaluation	Short‐term application	Skin smoothness, exfoliation, tolerability	Improved skin texture and superficial exfoliation with generally favorable tolerability	Limited controlled clinical trials; heterogeneous formulations	[46, 53]
Bromelain	Liposomal and niosomal topical formulations	Experimental dermatological and topical delivery studies	Short‐term	Collagen modulation, skin softening, topical tolerability	Enhanced stability and delivery efficiency in encapsulated systems	Mostly ex vivo or pilot‐scale studies; limited cosmetic clinical validation	[24, 25, 27]
Proteolytic enzyme blends (papain, bromelain, ficin)	Exfoliating skincare products	In vitro, ex vivo, and limited consumer‐oriented evaluations	Variable	Proteolytic activity, desquamation, skin compatibility	Demonstrated exfoliating activity and potential cosmetic applicability	Lack of standardized comparative clinical trials	[10, 28, 53]
Superoxide dismutase (SOD)	Liposomal or peptide‐assisted topical antioxidant systems	Human skin models and limited in vivo evaluations	Short‐term	Oxidative stress reduction, photoprotection, skin response to UV exposure	Reduced oxidative stress biomarkers and UV‐associated skin damage	Predominantly biomarker‐based outcomes; limited long‐term cosmetic studies	[15, 60, 64]
Catalase	Antioxidant co‐formulations	Experimental and dermatological oxidative stress models	Short‐term	Hydrogen peroxide reduction, oxidative stress markers	Demonstrated antioxidant activity and ROS detoxification potential	Limited direct cosmetic clinical evidence	[16, 55]
Coenzyme Q10 (CoQ10)	Creams, liposomal systems, nanoformulations	Human cosmetic studies and anti‐aging evaluations	Several weeks to months	Skin elasticity, wrinkle appearance, firmness	Improvements in skin smoothness, elasticity, and photoprotection‐related parameters	Variability among formulations and study methodologies	[47, 54]
Tyrosinase‐targeted depigmenting systems	Topical brightening formulations	Human hyperpigmentation and cosmetic studies	Variable	Pigmentation intensity, skin tone uniformity	Gradual reduction in hyperpigmentation and skin tone improvement	Heterogeneous assessment methods and limited long‐term follow‐up	[50, 65, 67]
Laccase‐mediated depigmenting systems	Enzyme‐assisted whitening systems	Experimental skin and melanin decolorization studies	Short‐term	Melanin degradation, whitening effect	Potential skin‐whitening activity through melanin modification	Primarily in vitro studies; minimal clinical validation	[83, 86, 87]

Abbreviations: ROS, reactive oxygen species; SOD, superoxide dismutase.

## 7 Conclusion

Enzymes represent a versatile and evolving class of cosmetic actives whose effectiveness is governed less by intrinsic catalytic properties and more by formulation‐dependent factors such as stability, delivery, and controlled activity within complex product matrices. Current evidence suggests that cosmetic enzymes may function more effectively as formulation‐integrated actives within engineered delivery systems. Comparative evaluation of microbial, fungal, and plant‐derived enzymes highlights complementary strengths, with enzyme selection increasingly determined by formulation context and intended performance rather than biological origin alone.

Advances in recombinant production, encapsulation, and carrier‐based delivery have significantly expanded the applicability of enzymatic systems in skincare. However, translation into consistent cosmetic efficacy remains limited by insufficient long‐term clinical validation, variability in evaluation methodologies, and unresolved challenges related to skin penetration and activity control. These gaps continue to constrain the establishment of robust functional claims and standardized performance benchmarks.

Future progress in enzyme‐based cosmetics will depend on integrating formulation science, biotechnology, and clinical research within a system‐level design framework. Future research should prioritize standardized enzyme activity assays, long‐term human clinical studies, formulation‐specific stability testing, and comprehensive safety evaluation under realistic storage and application conditions. Strengthening the link between mechanistic understanding, formulation architecture, and clinical outcomes will be essential for advancing enzymes from promising bioactive concepts to reliable, evidence‐based cosmetic technologies suitable for large‐scale industrial application.

## Conflicts of Interest

The authors declare no conflicts of interest.

## Data Availability

Data sharing not applicable to this article as no datasets were generated or analysed during the current study.
